# Supplementary material for: Field and Laboratory Observations on the Biology of Aceria angustifoliae with Emphasis on Emergence of Overwintering Mites
Source: Insects. 2023 Jul 13;14(7):633. doi: 10.3390/insects14070633 (PMC10380983; doi:10.3390/insects14070633)
Supplement: Supplementary file 1 [file insects-14-00633-s001.zip › insects-2426208-supplementary.pdf]

insects

Supplemental Material S1. Meteorological data of the experiment locality during December-March 2017-2018 and 2018-2019 (Taken from https://www.accuweather.com/); the days with temperature average above Tb marked.

Date		Temperature		
Month	Day	max	min	average
December 2017	1	10	-1	4.5
	2	9	-5	2
	3	9	-3	3
	4	7	-4	1.5
	5	8	-3	2.5
	6	14	-1	6.5
	7	5	-8	-1.5
	8	2	-12	-5
	9	4	-8	-2
	10	3	-7	-2
	11	4	-7	-1.5
	12	4	-6	-1
	13	4	-5	-0.5
	14	4	-5	-0.5
	15	5	-5	0
	16	5	-4	0.5
	17	6	-4	1
	18	6	-4	1
	19	7	-2	2.5
	20	11	0	5.5
	21	12	5	8.5
	22	11	2	6.5
	23	14	5	9.5
	24	14	7	10.5
	25	11	4	7.5
	26	9	-2	3.5
	27	9	-2	3.5
	28	9	-1	4
	29	11	0	5.5
	30	16	2	9
	31	13	2	7.5
January 2018	1	11	2	6.5
	2	9	1	5
	3	7	0	3.5
	4	6	-3	1.5
	5	7	-2	2.5

Date		Temperature		
Month	Day	max	min	average
December 2018	1	11	6	8.5
	2	11	5	8
	3	11	2	6.5
	4	10	1	5.5
	5	10	4	7
	6	8	4	6
	7	6	1	3.5
	8	7	3	5
	9	9	3	6
	10	8	0	4
	11	10	-2	4
	12	13	0	6.5
	13	11	4	7.5
	14	7	-2	2.5
	15	8	-2	3
	16	9	-1	4
	17	7	2	4.5
	18	3	1	2
	19	6	2	4
	20	4	0	2
	21	4	0	2
	22	7	-1	3
	23	5	-2	1.5
	24	6	-2	2
	25	6	0	3
	26	7	-3	2
	27	9	1	5
	28	8	-2	3
	29	0	-4	-2
	30	4	-1	1.5
	31	5	-4	0.5
January 2019	1	7	1	4
	2	5	-4	0.5
	3	1	-4	-1.5
	4	1	-1	0
	5	3	-3	0

	6	6	-2	2		6	8	-5	1.5
	7	9	-2	3.5		7	7	0	3.5
	8	7	-3	2		8	7	-3	2
	9	6	-4	1		9	7	-2	2.5
	10	7	-5	1		10	3	-6	-1.5
	11	7	-4	1.5		11	3	-7	-2
	12	11	-2	4.5		12	2	-7	-2.5
	13	10	-3	3.5		13	4	-7	-1.5
	14	11	-4	3.5		14	8	-1	3.5
	15	5	-2	1.5		15	9	-2	3.5
	16	4	-3	0.5		16	10	-2	4
	17	1	-7	-3		17	6	0	3
	18	6	-4	1		18	0	-5	-2.5
	19	11	1	6		19	-1	-8	-4.5
	20	5	1	3		20	1	-8	-3.5
	21	5	-3	1		21	1	-9	-4
	22	6	-2	2		22	2	-8	-3
	23	8	-3	2.5		23	3	-7	-2
	24	9	-1	4		24	4	-6	-1
	25	9	1	5		25	7	-3	2
	26	6	1	3.5		26	9	-2	3.5
	27	3	-2	0.5		27	10	-2	4
	28	-1	-6	-3.5		28	6	3	4.5
	29	0	-13	-6.5		29	6	0	3
	30	0	-9	-4.5		30	6	-1	2.5
	31	3	-10	-3.5		31	8	-2	3
February 2018	1	7	-6	0.5	February 2019	1	9	-2	3.5
	2	7	-6	0.5		2	8	-2	3
	3	7	-5	1		3	5	-4	0.5
	4	7	-4	1.5		4	7	-2	2.5
	5	11	-3	4		5	7	-4	1.5
	6	13	-1	6		6	7	-3	2
	7	14	-1	6.5		7	8	-2	3
	8	11	-1	5		8	13	1	7
	9	10	-2	4		9	7	0	3.5
	10	11	-3	4		10	8	2	5
	11	13	-2	5.5		11	6	0	3
	12	12	6	9		12	7	0	3.5
	13	6	3	4.5		13	8	-2	3
	14	8	2	5		14	9	-1	4
	15	10	0	5		15	11	2	6.5

	16	10	2	6		16	5	0	2.5
	17	8	4	6		17	5	-2	1.5
	18	8	0	4		18	6	-4	1
	19	3	-2	0.5		19	6	-2	2
	20	8	-2	3		20	6	-4	1
	21	10	3	6.5		21	8	-3	2.5
	22	7	3	5		22	8	-2	3
	23	9	4	6.5		23	9	-3	3
	24	7	3	5		24	10	-2	4
	25	9	0	4.5		25	10	-2	4
	26	7	1	4		26	11	-3	4
	27	10	4	7		27	10	4	7
	28	12	1	6.5		28	8	1	4.5
March 2018	1	12	1	6.5	March 2019	1	5	-2	1.5
	2	13	3	8		2	9	-4	2.5
	3	14	1	7.5		3	11	-2	4.5
	4	17	3	10		4	9	3	6
	5	16	9	12.5		5	10	2	6
	6	13	5	9		6	10	2	6
	7	15	5	10		7	10	-1	4.5
	8	18	4	11		8	7	1	4
	9	16	9	12.5		9	10	-1	4.5
	10	12	3	7.5		10	10	-1	4.5
	11	13	3	8		11	13	1	7
	12	14	5	9.5		12	14	0	7
	13	15	1	8		13	12	2	7
	14	16	3	9.5		14	16	4	10
	15	19	4	11.5		15	7	4	5.5
	16	16	8	12		16	12	2	7
	17	8	3	5.5		17	10	4	7
	18	14	0	7		18	7	1	4
	19	18	5	11.5		19	5	0	2.5
	20	20	5	12.5		20	6	2	4
	21	21	6	13.5		21	9	1	5
	22	18	5	11.5		22	12	0	6
	23	22	6	14		23	13	2	7.5
	24	24	8	16		24	13	1	7
	25	18	10	14		25	8	4	6
	26	19	7	13		26	11	0	5.5
	27	22	6	14		27	12	4	8
	28	22	10	16		28	10	4	7

	29	24	11	17.5		29	12	4	8
	30	19	10	14.5		30	14	6	10
	31	15	5	10		31	9	6	7.5
April 2018	1	16	3	9.5	April 2019	1	11	4	7.5
	2	17	3	10		2	12	5	8.5
	3	19	7	13		3	15	3	9
	4	16	9	12.5		4	14	4	9
	5	17	2	9.5		5	15	5	10
	6	19	7	13		6	16	4	10
	7	21	9	15		7	15	8	11.5
	8	21	5	13		8	17	6	11.5
	9	20	9	14.5		9	18	7	12.5
	10	21	8	14.5		10	19	6	12.5
	11	19	12	15.5		11	21	8	14.5
	12	19	9	14		12	18	12	15
	13	16	11	13.5		13	15	6	10.5
	14	17	9	13		14	16	3	9.5
	15	14	6	10		15	21	8	14.5
	16	11	3	7		16	18	12	15
	17	16	0	8		17	15	6	10.5
	18	19	4	11.5		18	16	3	9.5
	19	23	6	14.5		19	14	5	9.5
	20	21	6	13.5		20	9	4	6.5
	21	19	5	12		21	13	4	8.5
	22	12	3	7.5		22	7	2	4.5
	23	14	7	10.5		23	9	1	5
	24	19	5	12		24	12	0	6
	25	21	6	13.5		25	14	1	7.5
	26	22	10	16		26	15	3	9
	27	16	13	14.5		27	17	7	12
	28	20	9	14.5		28	18	5	11.5
	29	18	10	14		29	20	6	13
	30	17	10	13.5		30	20	9	14.5
